# Adaptive changes in adrenal steroid metabolism under extreme physical and psychological stress: a narrative review

**DOI:** 10.3389/fendo.2026.1860059

**Published:** 2026-07-01

**Authors:** Daria Borakowska, Rafał Podgórski, Edyta Łuszczki

**Affiliations:** 1 University of Information Technology and Management in Rzeszow, Rzeszów, Poland; 2 Faculty of Medicine, Collegium Medicum, University of Rzeszów, Rzeszów, Poland; 3 Faculty of Health Sciences and Psychology, Collegium Medicum, University of Rzeszow, Rzeszów, Poland

**Keywords:** adrenal steroid metabolism, cortisol, cortisone, DHEA-s, functional hypercortisolism, HPA axis, low energy availability, military stress

## Abstract

**Background:**

Chronic exposure to sleep deprivation, insufficient recovery, inadequate energy availability, extreme environmental conditions, repetitive high-intensity exercise, and psychological threat can alter hypothalamic-pituitary-adrenal (HPA) axis function. Acute activation of the HPA axis is adaptive, whereas prolonged or repetitive activation may contribute to allostatic load, altered daily glucocorticoid exposure, and changes associated with psychological and metabolic maladaptation.

**Methods:**

This narrative review synthesizes evidence on adrenal steroid metabolism under extreme physical and psychological stress, with emphasis on glucocorticoids, mineralocorticoids, and adrenocorticotropic hormone (ACTH)-responsive adrenal androgens, including dehydroepiandrosterone sulfate (DHEA-S) and androstenedione. PubMed and Google Scholar were searched through 22 May 2026. Recent human and military studies were prioritized, and earlier seminal work was included when it provided a mechanistic or historical context not captured by recent literature.

**Summary of evidence:**

The review describes how acute stress responses may support substrate mobilization and performance, whereas chronic exposure may flatten circadian patterns, alter ultradian glucocorticoid pulsatility, suppress downstream reproductive and metabolic axes, and produce heterogeneous effects on DHEA-S, aldosterone, and neurosteroid pathways. The strongest clinical and military evidence concerns sleep/circadian disruption, energy deficit, and prolonged military training; evidence for direct nutrient-mediated prevention of HPA-axis maladaptation remains preliminary or indirect.

**Conclusion:**

Addressing energy deficit, sleep deprivation and circadian disruption, inadequate recovery, dehydration/heat strain, and clinically relevant nutrient deficiencies may reduce the stressor load that contributes to functional hypercortisolism. Nutritional interventions should focus on correcting deficiencies, maintaining energy, supporting musculoskeletal function, and reducing stress, rather than serving as direct hormonal therapy. Future studies should use standardized steroid sampling, report sex-specific outcomes, and account for energy intake, expenditure, sleep, baseline fitness, and environmental exposures.

## 1 Introduction

Stress is a real or perceived challenge to homeostasis triggered by physical or psychological stimuli (1). The stress response coordinates neural, endocrine, immune, cardiovascular, and metabolic pathways, principally the sympatho-adrenal-medullary system and the hypothalamic-pituitary-adrenal (HPA) axis (1, 2). In acute settings, this response is adaptive because it mobilizes glucose and lipids, redistributes blood flow, and prioritizes vigilance and tissue protection (3). When stress is intense, repetitive, or prolonged, the same pathways may contribute to allostatic load, endocrine dysregulation, inflammation, sleep disruption, reproductive suppression, and impaired recovery (2, 4).

Military personnel, polar expedition members, endurance athletes, and other people exposed to austere environments provide human models of combined physical and psychological stress. Military training is especially relevant because energy deficit, sleep restriction, circadian disruption, heat or cold exposure, social hierarchy, occupational uncertainty, and threat perception often occur together. This multistressor setting makes it difficult to attribute endocrine changes to a single exposure, but it is highly translational because these stressors are rarely isolated in operational practice (5).

Testosterone, an anabolic hormone, aids muscle growth and repair, while cortisol, a catabolic hormone, is linked to stress responses and muscle breakdown (6). By analyzing the testosterone to cortisol balance, researchers can gain insights into an individual’s physiological state and identify potential imbalances affecting health and performance (7). The testosterone:cortisol ratio may be a useful tool for studies examining the relationships between hormonal levels and various physical and psychological outcomes. It can be useful when measured repeatedly with consistent timing and matrix, but it should not be overinterpreted because testosterone and cortisol have different rhythms, binding characteristics, and response kinetics (8, 9). Female military personnel warrant specific attention. In a 44-week infantry-based training study in women, Gifford et al. reported positive HPA-axis adaptation during training (6). Subsequent work in a related cohort demonstrated maladaptive reproductive and metabolic responses to chronic multistressor training, and a sex-comparative military training study showed different HPA and HPG-axis adaptation in women and men (6, 10). These data suggest that sex is not merely a demographic variable but a determinant of endocrine response, recovery, and vulnerability. The military socio-cultural environment can also add psychological stress through occupational norms, perceived evaluation, restricted autonomy, and exposure to gendered expectations; in such settings, female sex may become an avoidable physiological disadvantage when training and recovery systems are not appropriately designed (11).

PTSD and anxiety-related conditions are also relevant to military populations. PTSD is not best conceptualized as simple hypercortisolism: systematic reviews describe heterogeneous HPA-axis profiles, often including lower morning cortisol or blunted cortisol awakening response, increased evening/nadir cortisol in some cohorts, and strong effects of trauma type, comorbidity, medication, sleep disorder, and sampling method (12). Acute military stress activates the HPA axis, increasing cortisol to boost energy through glucose release. However, prolonged stress can cause burnout, lower testosterone, and alter HPA axis reactivity. Elevated cortisol negatively affects physical and mental health, with the risk of burnout increasing due to sleep deprivation, energy deficits, and extreme exertion (8). This heterogeneity supports a steroid-metabolism framework that distinguishes acute adaptive HPA activation from chronic dysregulation of timing, tissue exposure, receptor sensitivity, and local steroid metabolism (8, 12).

### 1.1 Steroid hormone characterization

Steroid hormones regulate growth, metabolism, salt and water balance, reproduction, immune function, and brain stress processing (13–16). They are synthesized from cholesterol through tissue-specific enzymatic pathways in the adrenal cortex, gonads, placenta, and selected peripheral tissues (16–18). The adrenal cortex produces mineralocorticoids in the zona glomerulosa, glucocorticoids in the zona fasciculata, and adrenal androgens mainly in the zona reticularis (18–20). Their biological activity depends not only on circulating concentrations but also on binding proteins, circadian and ultradian secretion, receptor availability, and tissue-specific activation or inactivation by steroid-metabolizing enzymes (17).

Androgens require more precise nomenclature than a generic “sex steroid” label. Testosterone and dihydrotestosterone are potent androgens that drive many male secondary sexual characteristics and contribute to muscle mass, erythropoiesis, bone size, libido, and performance-related physiology (9, 11). DHEA is better described as a preandrogen, whereas androstenedione is a weak androgen and androgen precursor. DHEA-S and androstenedione are produced substantially by the adrenal cortex, are exquisitely sensitive to ACTH compared with testosterone, and may provide biomarkers of adrenal adaptation or stress buffering (21–24). Their interpretation requires attention to age, sex, oral contraceptive use, menstrual status, circadian phase, acute workload, and assay method (21, 22).

### 1.2 Steroid biosynthesis, transport, and clearance

Steroidogenesis begins with cholesterol transport into mitochondria and conversion to pregnenolone by CYP11A1 (19, 20). Subsequent enzymatic steps involving HSD3B, CYP17A1, CYP21A2, CYP11B1, and CYP11B2 determine whether steroid precursors are directed toward glucocorticoid, mineralocorticoid, or androgen synthesis. ACTH is the major driver of zona fasciculata and zona reticularis steroidogenesis, whereas aldosterone synthesis is regulated primarily by the renin-angiotensin-aldosterone system (RAAS), plasma potassium, and local adrenal mechanisms, with ACTH providing an additional acute stimulus (16–20).

Circulating steroid concentrations are only part of the biological signal. Cortisol is transported mainly bound to corticosteroid-binding globulin and albumin; changes in binding proteins, inflammation, estrogen exposure, or energy deficit can alter total hormone concentrations without equivalent changes in free hormone exposure. Cortisol also exhibits circadian and ultradian pulses. Therefore, single untimed samples may misclassify HPA-axis activity (25).

Local steroid metabolism is central to adrenal endocrinology. 11β-hydroxysteroid dehydrogenase type 1 (11β-HSD1) regenerates cortisol from cortisone in the liver, adipose tissue, brain, and other tissues, amplifying glucocorticoid action locally. 11β-HSD2 converts cortisol to cortisone in mineralocorticoid-sensitive tissues such as the kidney, colon, placenta, and salivary glands, thereby protecting mineralocorticoid receptors from cortisol excess (16). Because salivary glands express 11β-HSD2, simultaneous measurement of salivary cortisol and cortisone can provide useful information about exposure and local conversion, although pre-analytical timing remains critical (25, 26).

**Figure 1:**
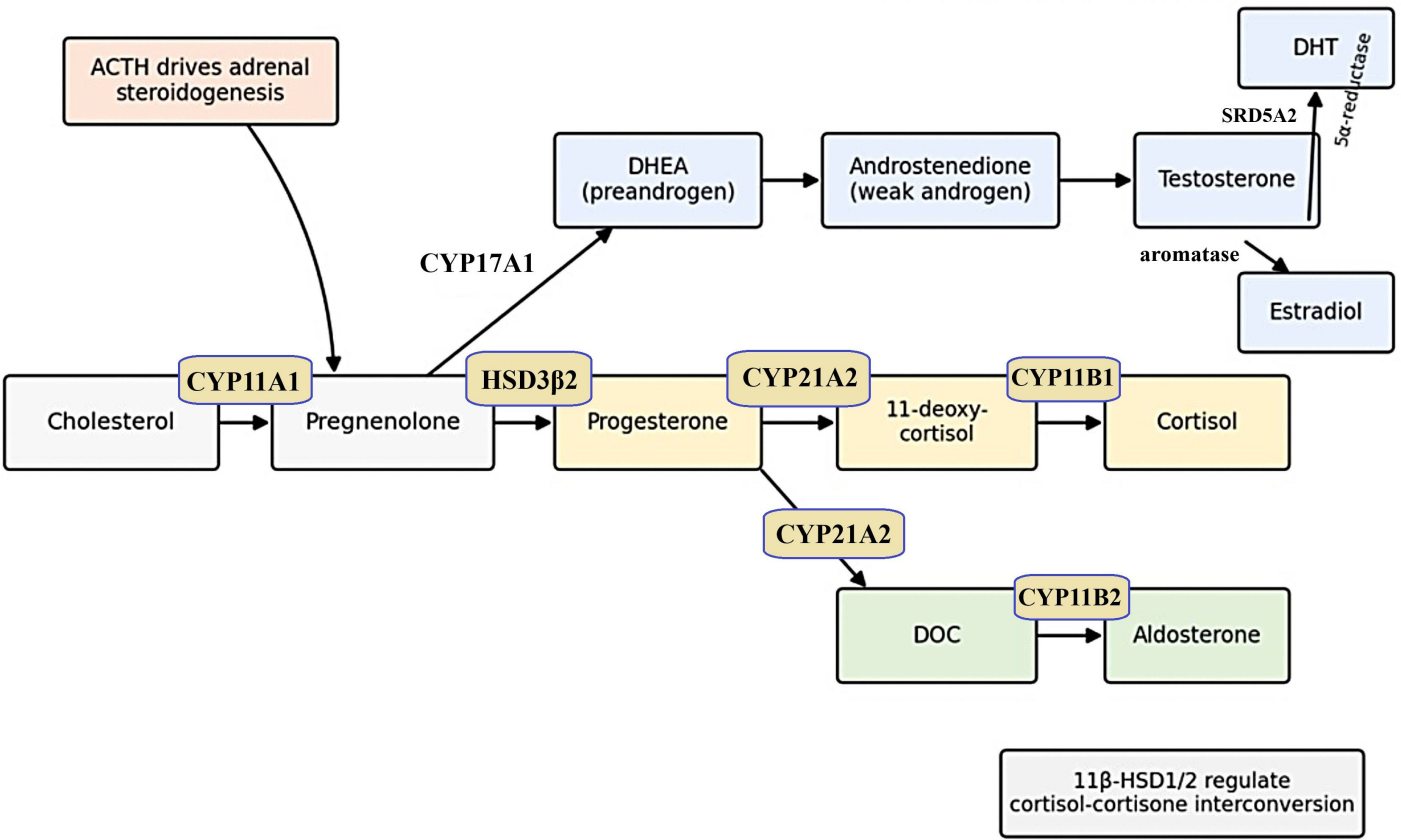
Simplified steroidogenic pathways relevant to stress physiology. SRD5A2, steroid 5-alpha-reductase 2; 11β-HSD1/2, 11β-hydroxysteroid dehydrogenase types 1 and 2; ACTH, adrenocorticotropic hormone; CYP11A1, P450 side-chain cleavage enzyme; CYP11B1, 11β-hydroxylase; CYP11B2, aldosterone synthase; CYP17A1, 17α-hydroxylase; CYP21A2, 21-hydroxylase; HSD3β2, 3β-hydroxysteroid dehydrogenase type 2; DOC, 11-deoxycorticosterone.

Other enzymes, including 5α-reductases, aromatase, sulfotransferases, sulfatases, and glucuronosyltransferases, further shape steroid activity and clearance. These pathways may be altered by inflammation, nutrition, adiposity, liver function, medication, sex, and chronic stress, but human operational data remain limited and heterogeneous (27–29). **Figure 1** summarizes simplified steroidogenic pathways relevant to stress physiology, with emphasis on adrenal glucocorticoids, mineralocorticoids, and ACTH-responsive adrenal androgens, while also acknowledging downstream gonadal androgen and estrogen pathways.

### 1.3 Scope, materials, and methods

This narrative review synthesizes evidence on adrenal steroid metabolism under conditions of extreme physical and psychological stress, focusing on glucocorticoids, mineralocorticoids,ACTH-responsive adrenal androgens, including DHEA-S and androstenedione, and clinically relevant interactions with gonadal, neurosteroid, metabolic, and inflammatory pathways. Therefore, unless otherwise specified, the term “steroids” is used in the context of adrenal endocrinology.

PubMed and Google Scholar were searched through 22 May 2026 using combinations of the following terms: “cortisol”, “cortisone”, “glucocorticoids”, “HPA axis”, “DHEA-S”, “dehydroepiandrosterone sulfate”, “androstenedione”, “aldosterone”, “mineralocorticoid”, “military training”, “special forces”, “sleep deprivation”, “circadian disruption”, “energy deficit”, “low energy availability”, “hypoxia”, “altitude”, “heat stress”, “cold exposure”, “PTSD”, “depression”, “anxiety”, “neurosteroids”, “vitamin D”, “magnesium”, “omega-3”, “leucine”, and “alpha-lipoic acid”. Human studies, military/operational cohorts, controlled laboratory studies, and high-quality reviews were prioritized. Earlier seminal studies were added where they remain foundational for stress physiology, sleep-HPA interactions, or military endocrine adaptation (3, 21, 22, 30, 31).

## 2 Adaptive adrenal steroid remodeling under extreme physical and psychological stress

### 2.1 Sleep disruption, circadian misalignment, and glucocorticoid timing

Prolonged stress is linked to poor sleep quality and various disturbances, such as insomnia, difficulty falling asleep, and increased daytime sleepiness (32). Sleep issues can lead to serious health problems like obesity, hypertension, and cardiovascular disease, which are particularly common in military populations (32). Soldiers often face sleep disorders due to harsh conditions, operational stress, and long duty hours. Insufficient sleep significantly impairs performance, reducing combat effectiveness by 15–25% for each lost hour, with extreme cases showing soldiers functioning at only 15% effectiveness after just 4 hours of sleep (33).

Sleep deprivation also disrupts hormone secretion, particularly cortisol and testosterone. Night shift work affects the circadian rhythm, altering cortisol patterns and leading to hormonal desynchronization (34, 35). While a meta-analysis showed no significant overall increase in cortisol due to sleep deprivation, important effects were noted in specific studies (36). In young men, just one week of restricted sleep reduced testosterone levels by 10–15% (37). Additionally, sleep deprivation lowers the nighttime rise in aldosterone, influenced by both the sleep–wake cycle and circadian rhythms (38, 39). Sleep deprivation and circadian misalignment are among the most reproducible operational stressors affecting glucocorticoid regulation. Seminal work by Leproult et al. showed that sleep loss elevates cortisol the following evening, and Spiegel et al. linked sleep debt with endocrine and metabolic impairment (30, 31). These studies remain important because later military and endurance studies often combine sleep loss with energy deficit, physical workload, and psychological stress, making causal isolation difficult.

Cortisol secretion is organized by both circadian and ultradian mechanisms (25). The suprachiasmatic nucleus (SCN) entrains the daily rhythm, while sub-hypothalamic and pituitary-adrenal feedback mechanisms generate pulsatile ACTH-cortisol output. This means that “increased cortisol secretion” must be specified as an acute stress response, a higher 24-hour exposure, an altered cortisol awakening response, an evening elevation, a flatter diurnal slope, or disrupted pulse dynamics (25). Glucocorticoids interact with central and peripheral clocks. The SCN coordinates light-entrained rhythms, while peripheral clocks in liver, muscle, adipose tissue, immune cells, and adrenal tissue respond to feeding, activity, temperature, and glucocorticoid timing. Ultradian cortisol pulses provide dynamic information to target tissues; flattening or mistiming of these pulses may impair receptor signaling even when a single cortisol value appears normal (25).

Sleep restriction and circadian disruption may interact with RAAS activity, sympathetic tone, blood pressure regulation, appetite, insulin sensitivity, and inflammatory signaling (40). Sleep interventions are therefore discussed as operational countermeasures rather than endocrine therapies. Sleep extension before anticipated deprivation, protected sleep windows, scheduled/rotational naps, and circadian light management can improve performance and may support more stable HPA-axis rhythmicity, but field trials with steroid outcomes remain limited (40, 41).

### 2.2 General stress response and adrenal steroid homeostasis

Stress directly influences adrenal steroid homeostasis through ACTH, autonomic activation, inflammatory mediators, RAAS, and tissue-level steroid metabolism. Selye’s general adaptation syndrome remains a useful historical framework: acute alarm responses can be protective, sustained resistance requires metabolic allocation, and chronic overload may lead to exhaustion-like maladaptation (3). Modern physiology refines this model by emphasizing allostasis, receptor sensitivity, timing of secretion, and local steroid metabolism rather than a simple linear increase in cortisol.

In acute stress, ACTH pulses stimulate cortisol and adrenal androgen precursors, while RAAS and sympathetic activation support blood pressure and fluid balance. Cortisol mobilizes energy substrates and constrains excessive inflammation (24, 42). DHEA-S and androstenedione may increase with ACTH and have been proposed as markers of adrenal reserve or stress buffering, although the magnitude and direction of change vary by sex, age, training status, timing, and stressor type (23, 24). In chronic stress, evidence is more heterogeneous: some cohorts show elevated integrated cortisol exposure, whereas others show reduced morning cortisol, flatter diurnal slopes, or blunted responses, particularly in trauma-related conditions and after prolonged training (7, 21, 26).

### 2.3 Depression, anxiety, PTSD, and mineralocorticoid signaling

Psychiatric disorders are relevant to adrenal endocrinology because they alter both central stress appraisal and peripheral steroid action. Major depressive disorder has been associated with hypercortisolemia, impaired negative feedback, altered glucocorticoid receptor sensitivity, and changes in mineralocorticoid receptor (MR) function, but these patterns are not universal (43–45). Anxiety disorders and PTSD add further heterogeneity: PTSD frequently involves altered diurnal timing, blunted cortisol awakening or stress responses in some studies, and increased evening/nadir cortisol in others (12).

A systematic review and meta-analysis by Sahu et al. found that serum and plasma cortisol levels are typically elevated in patients with depression compared to healthy controls (43). However, variations in cortisol levels depend on depression phenotype, symptom severity, chronicity, and sampling protocol, potentially limiting the generalizability. This pattern suggests persistent stress system activation and impaired negative feedback, contributing to emotional dysregulation, sleep issues, cognitive impairment, and increased metabolic stress (43). DHEA-S levels are often lower in depression, indicating a shift toward a less resilient state when combined with glucocorticoid excess (46). Meta-analysis indicates that DHEA supplementation may have modest antidepressant effects for some patients (47).

Aldosterone and mineralocorticoid receptor dysfunction are clinically relevant but less consistently documented than cortisol or neurosteroids (48, 49). Nevertheless, Young et al. and Murck et al. suggest altered mineralocorticoid receptor function in major depression and an increased burden of depressive and anxiety symptoms in primary aldosteronism (44, 45, 50). These findings suggest that depression involves broader dysregulation of multiple hormonal pathways, including glucocorticoid, adrenal androgen, gonadal, mineralocorticoid, and neurosteroid systems, which may serve as biomarkers and therapeutic targets.

MR dysfunction is clinically relevant because MR signaling contributes to appraisal, memory, salt appetite, blood pressure, and stress resilience. Primary aldosteronism and other mineralocorticoid-excess states have been linked with mood and cognitive symptoms, but extrapolation from endocrine disease to functional stress states should be cautious (50, 51). This distinction is important when interpreting MR-mediated findings in stress-related disorders, because endocrine disease models cannot be assumed to represent reversible operational stress adaptations.

### 2.4 Glucocorticoid signaling, inflammation, and tissue injury

Glucocorticoids are anti-inflammatory in acute pharmacologic and physiologic contexts, but chronic or repetitive stress can produce more complex outcomes, including altered cytokine profiles, glucocorticoid receptor resistance, impaired tissue repair, and susceptibility to infection (2, 52). In operational settings, the relevant question is how repeated HPA-axis activation changes receptor sensitivity, local metabolism, and downstream immune signaling over time. Haley et al. highlighted the significant role of stress in increasing stroke risk and contributing to atherosclerosis, mainly through localized inflammation (53). Chronic low-grade systemic inflammation also plays a key role in the initiation and progression of atherosclerotic plaques in areas of minor arterial damage (54).

The main translational issue is that chronic glucocorticoid exposure may alter muscle protein turnover, visceral adiposity, insulin sensitivity, bone turnover, mood, cognition, and immune defense. These outcomes overlap with sleep loss and low energy availability, so causal attribution to cortisol alone is often not possible without careful study design.

### 2.5 Prolonged high-intensity training, military field studies, and steroid biomarkers

Cortisol is widely used as a biomarker of stress and exercise, but interpretation depends on the biological matrix and sampling design. Plasma or serum cortisol can capture acute endocrine responses but is affected by venipuncture stress and binding proteins. Salivary cortisol estimates free cortisol and is practical in the field, while salivary cortisone may be useful where 11β-HSD2 activity or contamination affects cortisol. Hair cortisol may index longer-term exposure but has limited temporal precision and is affected by hair treatment, growth rate, ethnicity, and environmental contamination. Urinary steroid profiling can capture production and metabolism, but is logistically demanding in field settings (25, 26).

Field studies are difficult because steroid sampling can interfere with training, and protocol adherence may be poor under operational conditions. In a British infantry training pilot study, carefully timed cortisol awakening response sampling was feasible only in a minority of planned samples, illustrating why robust field endocrinology requires simplified protocols, participant training, and realistic compliance expectations (26). Tait et al. studied Australian Army combat engineers during an 8-day military training period embedded in a 16-day protocol and observed changes in salivary cortisol, testosterone, sleep, and recovery dynamics, including incomplete recovery of the bedtime testosterone:cortisol ratio after training (7). Such work demonstrates that HPA and HPG-axis changes are time-dependent and that recovery windows matter as much as peak stress responses.

Sex-specific military data have expanded substantially. The Female Endocrinology in Arduous Training work demonstrated both positive HPA-axis adaptation during prolonged infantry training and subsequent reproductive/metabolic consequences in women (6, 55). A sex-comparative study reported different HPA and HPG-axis adaptation in men and women during military training (10). These findings support sex-stratified reporting and discourage extrapolating male-only data to female soldiers.

Extreme expedition studies also illustrate adaptation rather than simple pathology. In the all-female Ice Maiden Antarctic traverse, high workload, cold exposure and energy stress were accompanied by adrenal and metabolic changes, but careful preparation helped avoid the most severe maladaptive patterns (56). The INSPIRE22 ski traverse of Antarctica found preserved circadian variation in cortisol and androgens despite prolonged cold, exercise and energy challenge, with transient downstream endocrine suppression (57). These studies suggest that comprehensive preparation, sufficient energy provision, and recovery planning can convert some extreme exposures into manageable adaptive challenges (56, 57).

### 2.6 Extreme environmental conditions

Heat, cold, hypoxia, altitude, and mountainous terrain can all influence adrenal steroid signaling. Controlled laboratory studies isolate single stressors but may lack ecological validity; expedition and military studies are ecologically strong but usually have small samples, limited control groups, mixed sex distribution, and concurrent sleep and energy stress (56–60). Therefore, environmental conclusions should be framed as context-dependent rather than universal.

Hydration and heat-strain management are operationally important for thermoregulation, cardiovascular stability, renal perfusion, and perceived exertion. Hydration status may secondarily influence inflammatory and endocrine responses during humid heat exposure, although direct evidence for cortisol-mediated cytokine modulation in military settings remains limited (60). In heat, cortisol responses are influenced by core temperature, exercise intensity, hydration, and psychological strain (59, 60). In cold, endocrine adaptation depends on shivering/non-shivering thermogenesis, energy availability, catecholamine tone, sleep, and clothing/shelter (56–58). Hypoxia and altitude can activate HPA and sympathetic pathways, but acclimatization, illness, exertion, and energy deficit confound interpretation (59, 60). Future studies should report environmental exposure, energy balance, sleep, menstrual or hormonal contraceptive status, and sample timing in greater detail.

### 2.7 Energy deficit, fasting, calorie restriction, and low energy availability

Energy deficit is a critical mediator of endocrine adaptation under extreme stress. The recent review by Friedl et al. emphasized that stress-associated testosterone suppression often reflects central, reversible adaptation rather than intrinsic testicular failure, with energy deficit, sleep disruption, and psychogenic stress suppressing GnRH/LH signaling, lowering androgen output, and increasing sex hormone-binding globulin (9). This framework is important because inappropriate labeling as pathological hypogonadism could lead to unnecessary androgen therapy rather than correction of the operational stressor load.

Earlier military studies remain foundational. Opstad’s Norwegian Ranger work showed that prolonged physical stress combined with sleep and energy deficiency can extinguish normal circadian hormone rhythms (21). Friedl et al. demonstrated endocrine markers of semistarvation in lean men exposed to a multistressor environment, with testosterone, triiodothyronine, and IGF-1 among the affected axes (22). These findings align with contemporary concepts of low energy availability and relative energy deficiency, while reinforcing that adrenal, gonadal, thyroid, and growth-factor axes should be interpreted together.

**Figure 2:**
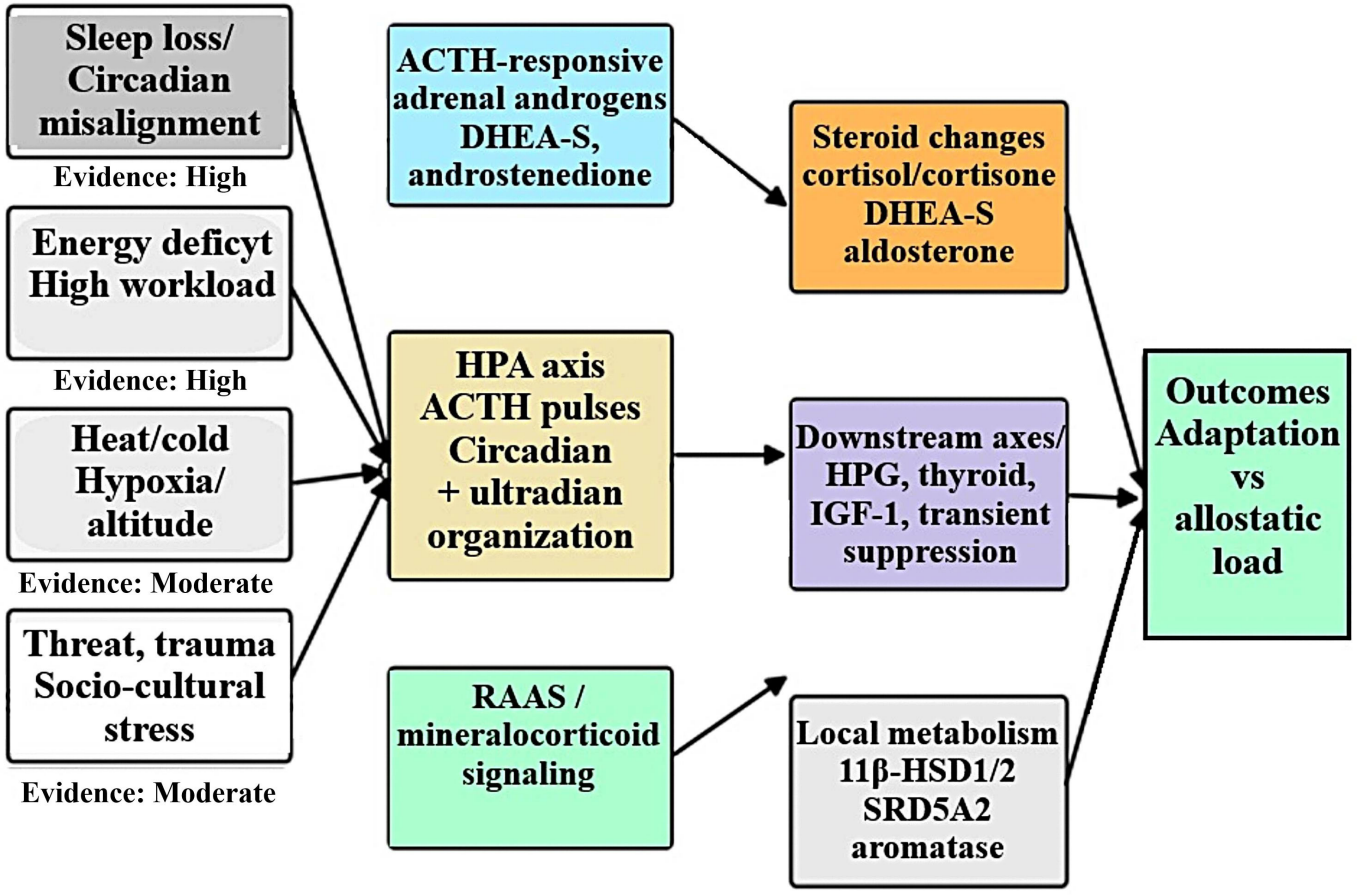
Integrative pathway linking operational stressors to steroid remodeling (7, 58–72). Directional arrows indicate plausible pathways linking operational stressors, adrenal steroid axes, local steroid metabolism, and outcomes. Evidence levels are shown to distinguish established operational findings from mechanistic inference. 11β-HSD1/2 - 11β-Hydroxysteroid Dehydrogenase Type 1 and Type 2; ACTH - Adrenocorticotropic Hormone; DHEA-S - Dehydroepiandrosterone Sulfate; HPA axis - Hypothalamic-Pituitary-Adrenal Axis; HPG - Hypothalamic-Pituitary-Gonadal Axis; IGF-1 - Insulin-Like Growth Factor 1; RAAS - Renin-Angiotensin-Aldosterone System; SRD5A2 - Steroid 5-Alpha-Reductase 2.

In women, low energy availability may present with hypothalamic menstrual disturbance, altered luteal function, impaired bone health, and changes in cortisol dynamics (10, 55). However, military studies indicate that energy availability alone may not explain all sex differences; psychosocial stress, occupational environment, sleep, body composition, contraceptive use, and baseline endocrine status also contribute (10, 11, 55). **Figure 2** summarizes plausible pathways linking operational stressors, including energy deficit, with adrenal steroid remodeling.

### 2.8 Neurosteroids and local steroid metabolism

Neurosteroids are steroids synthesized *de novo* in the nervous system or generated locally from circulating precursors in neural tissue. They include metabolites such as allopregnanolone, pregnanolone, DHEA, DHEA-S, and other compounds that modulate GABAergic, glutamatergic, and stress-related signaling. Neurosteroids are relevant because stress, sleep loss, depression, PTSD, and sex hormone changes can modify neuroactive steroid tone and thereby influence anxiety, arousal, cognition, and resilience (73). The evidence for neurosteroid pathways under military or extreme physical stress remains more mechanistic than clinically established. Agis-Balboa et al. and Maguire et al. point to reduced expression of type I 5α-reductase in human depression, while Frau et al. and Maguire et al. report similar findings in animal stress models (73–75). Together, human and animal studies suggest possible mechanisms; however, the small sample sizes and variations between animal and human models make it difficult to interpret these results clearly or apply them in clinical practice. Human studies are fewer, often small, and typically measure peripheral concentrations rather than central nervous system synthesis. Therefore, neurosteroid mechanisms should be presented as plausible pathways requiring further translational validation.

## 3 Therapeutic and preventive perspectives to reduce stressor load and improve resilience

The existing literature distinguishes between operationally implemented remedial measures and theoretical or early-stage strategies. Current data do not provide clear evidence that supplementation with vitamin D (76–80), magnesium (80–83), omega-3 fatty acids (84–86), leucine (87), potassium (82, 88), zinc (78, 79), or alpha-lipoic acid effectively prevents abnormal adaptation of the HPA axis in military personnel subjected to extreme stress conditions (88, 89). Consequently, nutritional interventions should primarily be regarded as a means to rectify deficiencies, maintain adequate energy levels, support musculoskeletal function, and mitigate the indirect effects of stress, rather than as a form of direct hormonal therapy (84, 86, 88).

Energy availability is the first priority. Adequate total energy, carbohydrate availability during high workload, and sufficient high-quality protein help reduce the need for catabolic adaptation and support recovery. Leucine, an amino acid found in whey protein, especially in cow’s milk products like natural yogurt and low-fat cottage cheese, can stimulate muscle protein synthesis, help preserve lean body mass, and reduce muscle breakdown. However, this does not constitute evidence that leucine directly treats hypercortisolism (87). Similarly, vitamin D is clinically important for bone and muscle by promoting intestinal absorption of calcium, phosphorus, and magnesium when deficient, yet extraskeletal HPA-axis effects remain largely unproven in humans (80, 90, 91). Magnesium and potassium should be corrected when deficient or clinically indicated, but routine use as HPA-axis modifiers is not established (92). Hypercortisolism boosts mineralocorticoid receptor activity in the renal tubules, increasing sodium absorption and potassium elimination (93). Conversely, magnesium deficiency can lead to higher potassium excretion by removing the blockade of the renal outer medullary potassium channel, causing hypokalemia (94). Additionally, potassium is responsible for the oxidative capacity of the 11βHSD2 enzyme, which converts cortisol to cortisone (95).

Omega-3 fatty acids may influence inflammation and stress-reactivity pathways, but available evidence should be interpreted as indirect in relation to military HPA adaptation (84, 85). Long-chain fatty acids, especially omega-3s like docosahexaenoic acid from fish oil and alpha-linolenic acid from flaxseed oil, may improve vitamin D bioavailability by promoting mixed intestinal micelle formation, which solubilizes lipophilic compounds like cholecalciferol more effectively than medium-chain fatty acids in coconut oil (86, 96). Alpha-lipoic acid has antioxidant and neuroprotective plausibility and preclinical stress-model data, but operational human trials demonstrating protection against maladaptive steroid remodeling have not been identified (97).

Sleep interventions may be more directly actionable. Protected sleep opportunity, sleep banking before unavoidable deprivation, scheduled daytime naps, and light/dark timing can improve alertness and performance and may support cortisol rhythmicity (40, 41). These interventions should be evaluated in pragmatic military studies that measure endocrine outcomes, cognitive performance, injury, mood, and operational feasibility.

Corticosterone supplementation is not recommended as a mitigation strategy in humans. Corticosterone is the dominant glucocorticoid in many rodent models, whereas cortisol is the principal human glucocorticoid (98). Using exogenous glucocorticoids outside of established indications, such as adrenal insufficiency, can worsen HPA-axis suppression, raise the risk of infections, contribute to osteoporosis (particularly due to decreased intestinal calcium absorption), and elevate blood pressure and increase the risk of lipid disorders (99, 100). For these reasons, corticosterone supplementation is not appropriate as a preventive intervention in healthy stress-exposed personnel.

**Figure 3:**
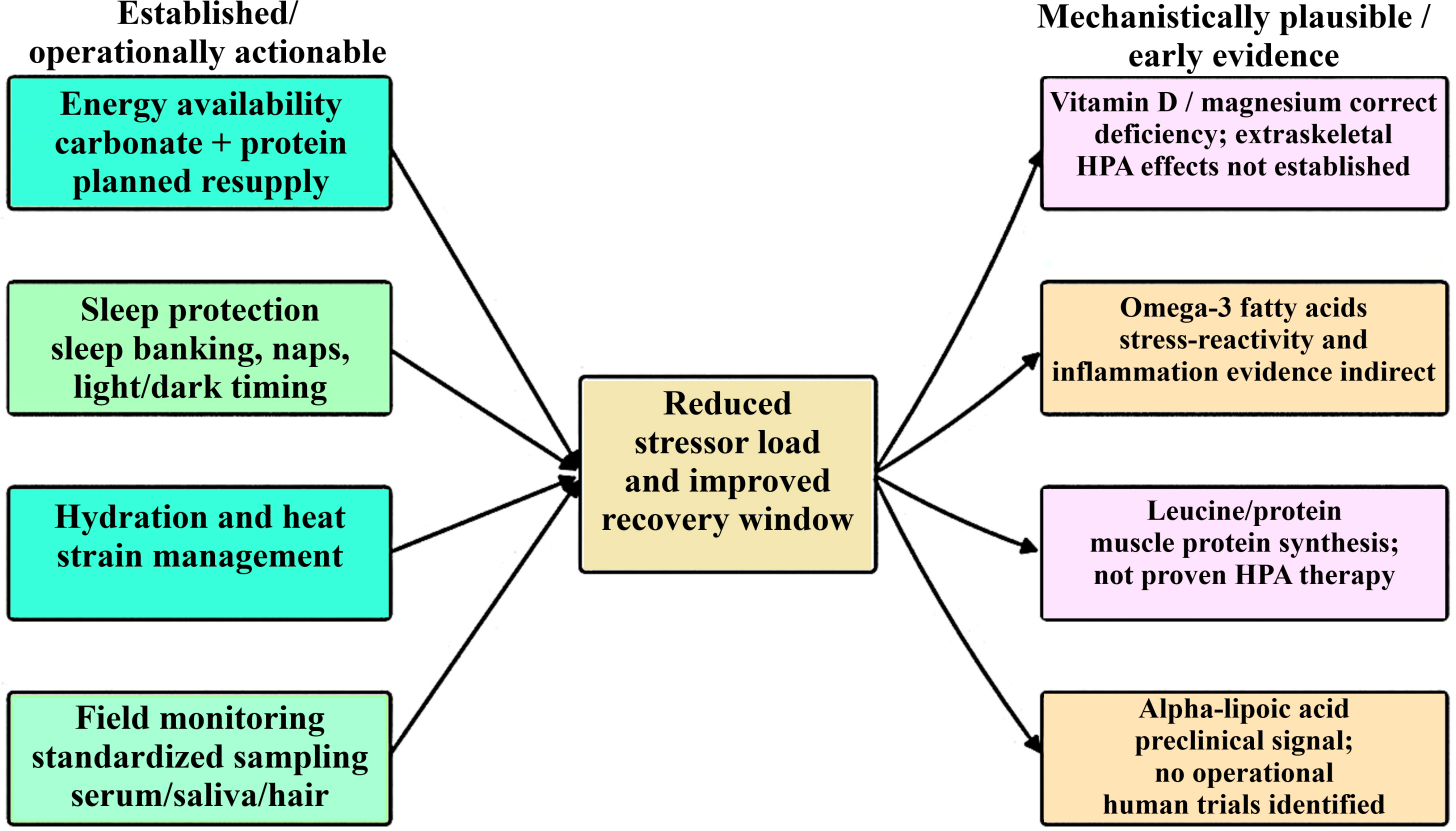
Countermeasures: established operational targets versus mechanistic plausibility (76, 77, 80–87, 89, 91, 94–96, 100, 102–108).

Monitoring should be individualized. A single cortisol value rarely determines clinical decision-making (101). Repeated measures with standardized timing, validated assays, and concurrent assessment of sleep, energy intake, workload, illness, medications, sex hormone status, and psychological symptoms are more informative (25, 26). Hair cortisol or urinary steroid profiling may complement salivary or serum sampling in selected studies, but should not replace clinical assessment (29). These distinctions are summarized in **Figure 3**, which distinguishes established, operationally actionable countermeasures from mechanistically plausible or early-evidence strategies.

**Table 1** presents the acute adaptive and chronic maladaptive endocrine responses to extreme stress, along with evidence and caveats for assessment. **Table 2** illustrates the strength of evidence alongside the principal limitations, organized by topic area.

**Table 1:** Acute adaptive versus chronic maladaptive endocrine responses to extreme stress (23, 24, 42, 73, 99, 108–119).

Domain	Acute adaptive response	Chronic/repeated maladaptive pattern	Evidence appraisal and caveat
HPA axis/glucocorticoids	ACTH-cortisol pulses mobilize glucose and lipids, support vigilance, and constrain excessive inflammation.	Higher integrated exposure, blunted morning response, flattened diurnal slope, altered ultradian dynamics, or functional hypercortisolism depending on context.	Strong physiological basis; human field data are heterogeneous and require standardized sampling.
Adrenal androgens (DHEA-S, androstenedione)	ACTH-responsive increase may accompany adrenal activation and potentially buffer stress.	Variable suppression or altered DHEA-S:cortisol balance with chronic stress, aging, sex differences, and energy deficit.	Moderate mechanistic evidence; biomarker role promising but not diagnostic.
RAAS/aldosterone/MR	Supports vascular tone, salt-water balance and acute adaptation to heat, dehydration or posture.	Hypertension, altered MR signaling, mood/cognitive effects in endocrine disease; functional stress data less certain.	Clinically strong in endocrine disorders; weaker for sleep/stress-only claims.
HPG axis/testosterone and estradiol	Short-term redistribution of energy away from reproduction may be adaptive.	Central suppression of testosterone, ovulatory dysfunction, luteal disturbance, increased SHBG, impaired bone and muscle adaptation.	Strong operational evidence for energy deficit and prolonged training; sex-stratified data are still sparse.
Neurosteroids	Modulation of arousal, anxiety and inhibitory/excitatory balance.	Potential alteration in PTSD, depression, sleep loss and chronic stress.	Mechanistically plausible; fewer operational human studies.
Metabolic and performance outcomes	Substrate mobilization and short-term performance readiness.	Insulin resistance, visceral adiposity, muscle loss, injury risk, osteoporosis (vitamin D deficiency), hypertension, hyperlipidemia, impaired recovery, mood, and cognitive effects.	Multifactorial; cortisol alone rarely explains the phenotype.

**Table 2:** Strength of evidence and major limitations by topic area.

Topic area	Main evidence base	Key limitations	Practical implication
Sleep and circadian disruption	Seminal laboratory studies; military sleep reviews; limited field endocrine studies.	Cortisol timing, prior sleep, caffeine, workload, and light exposure often not standardized.	Treat sleep as a modifiable operational risk factor; avoid overstating electrolyte consequences.
Energy deficit/low energy availability	Military semistarvation, Ranger, training and expedition studies; recent mechanistic review.	Concurrent sleep loss and workload complicate causal attribution.	Energy sufficiency is central to preventing downstream endocrine suppression.
Sex-specific responses	Female infantry studies, Antarctic traverse data, recent sex-comparative military training studies.	Small female samples in many studies; contraceptive and menstrual data often incomplete.	Report sex-stratified outcomes and design recovery/energy strategies for female personnel.
Environmental extremes	Heat/cold/hypoxia laboratory studies plus small expedition cohorts.	Small samples, limited controls, overlapping stressors.	Report exposure dose, acclimatization, sleep and energy variables.
Nutrition/supplements	Human nutrient trials, sports nutrition data, and preclinical stress models.	Few/no trials show prevention of maladaptive HPA-axis adaptation in military settings.	Correct deficiencies and ensure protein/energy adequacy; present supplement claims cautiously.
Neurosteroids/local metabolism	Mechanistic and translational literature; few operational cohorts.	Peripheral measures may not reflect central synthesis.	Frame as plausible mechanism requiring validation.

## 4 Limitations of this narrative review

This review has limitations inherent to its narrative design. It was not registered prospectively, did not use duplicate screening, did not formally grade risk of bias, and did not calculate pooled effect sizes. Selection bias is possible, especially because military and expedition endocrinology includes small, heterogeneous cohorts and because negative or logistically unsuccessful field studies may be underreported.

The included studies vary substantially in sex distribution, age, training status, environment, stressor duration, sleep opportunity, energy availability, medication exposure, hormonal contraceptive use, menstrual-cycle phase, sampling matrix, sample timing, and assay method. These differences limit direct comparison across studies and make it difficult to distinguish HPA-axis activation from altered local metabolism, binding proteins, or downstream receptor sensitivity.

The nutritional and neurosteroid sections rely partly on preclinical or translational evidence. These mechanisms are biologically plausible, but they should not be interpreted as clinically established interventions unless supported by human trials in relevant populations. Sex-specific evidence is improving but remains insufficient, particularly for female military personnel in combat-relevant environments.

## 5 Conclusions

Extreme physical and psychological stress produces adaptive changes in adrenal steroid metabolism that are context-dependent and time-sensitive. Acute HPA-axis activation supports survival and performance, but chronic or repeated stress may alter circadian and ultradian glucocorticoid signaling, suppress downstream reproductive and metabolic axes, and interact with inflammation, local steroid metabolism, and neurosteroid pathways. Cortisol remains a useful biomarker, but it should be interpreted alongside cortisone, DHEA-S, androstenedione, aldosterone-related variables, sleep, energy availability, sex, sample timing, and assay method.

The evidence synthesis supports a practical hierarchy: first address energy availability, sleep/circadian protection, recovery, environmental strain, hydration, and clinically documented nutrient deficiencies; then test mechanistically plausible interventions in carefully designed human studies. Future research should use standardized endocrine sampling, include sex-stratified analyses, report menstrual and contraceptive variables, and integrate endocrine outcomes with operational performance, health, and recovery.
